# Reproductive Ageing: Declining translational capacity as a potential driver for oocyte meiotic instability

**DOI:** 10.1530/REP-24-0198

**Published:** 2024-09-21

**Authors:** Katie J Danielson, Kayla L Judson, Ethan J Greenblatt

**Affiliations:** 1 Department of Biochemistry and Molecular Biology, University of British Columbia, Health Sciences Mall, Vancouver, British Columbia, Canada

## Abstract

**In Brief:**

This point of view article focuses on the potential contribution of defects in protein synthesis (translation) to the incidence of oocyte meiotic failure. We discuss the potential cause of diminished oocyte translation during aging and the impact of these deficits on the function of the meiotic spindle.

**Abstract:**

Errors during female meiosis lead to embryonic aneuploidy and miscarriage and occur with increasing frequency during aging. The underlying molecular changes that drive female meiotic instability remain a subject of debate. Developing oocytes undergo a tremendous increase in cytoplasmic volume over several months of follicle development and rely on long-lived mRNAs and ribosomes accumulated during this growth phase for subsequent meiotic maturation. In this point of view article, we discuss how the unique reliance on stores of long-lived mRNAs and ribosomes may represent an Achilles' heel for oocyte function and how alterations that reduce the translational capacity of oocytes could be a factor significantly contributing to female infertility. Understanding these mechanisms could lead to new therapeutic strategies to improve fertility outcomes.

Errors in chromosome segregation during female meiosis are a major cause of the high rate of miscarriage, estimated to occur at a frequency of 30–70%. Traditionally, it has been assumed that these errors arise because the linkages between sister chromatids, called cohesion, weaken over the several decades that dormant oocytes are stored within primordial follicles (Chiang *et al.* 2010, Lister *et al.* 2010, Webster & Schuh 2017). However, recent studies of *Drosophila* and mouse oocytes suggest that damage to the cytoplasmic contents of late-stage oocytes also contributes substantially. Oocytes cease transcribing RNA just prior to meiotic maturation, the process whereby diplotene-arrested oocytes progress to meiotic metaphase II in preparation for fertilization (Lodde *et al.* 2008). Instead, meiotic maturation relies on the utilization of cytoplasmic mRNAs and ribosomes that were previously accumulated during the extended period of oocyte growth that follows primordial follicle activation. In this perspective article, we hypothesize this unique reliance of late-stage oocytes on extremely long-lived mRNAs, tRNAs, and ribosomes makes them especially error prone. Defects in oocyte translation thus may represent a critical and underappreciated factor contributing to embryonic aneuploidy and infertility.

Human primordial follicles are formed during the third trimester of fetal development, establishing the lifetime ovarian reserve at birth. Once formed, primordial follicles are continuously activated into pools of growing follicles, but only progress past the secondary follicle stage in a gonadotropin-dependent fashion after puberty. Oocytes eventually grow into the largest cells in the body, undergoing a massive ~100-fold increase in cytoplasmic volume. In order to support such an extreme level of growth, growing oocytes transcribe RNA at high rates. In the growing oocytes of some amphibian species, the chromosomes are near-maximally packed with RNA polymerase II to their physical limit on transcribed genes, developing a characteristic ‘lampbrush’ morphology (Gall 2012). Whereas mRNAs typically turnover with half-lives on the order of ~7 h in dividing cells (Sharova *et al.* 2009), pulse-labeling of growing mouse oocytes showed that mRNA and ribosomal RNA molecules produced during these stages are surprisingly stable. Actively translated polysomal oocyte mRNAs had half-lives of ~6 days, while stored non-polysomal mRNAs and ribosomal rRNAs appeared not to be degraded at all, allowing for these molecules to accumulate (De Leon *et al.* 1983). As follicle growth reaches completion, transcription by the oocyte becomes entirely or nearly completely suppressed and the oocyte chromosomes become highly condensed around the nucleolus. Transcription remains repressed until zygotic genome activation in the early embryo at the eight (human) or two (mouse) cell stage. Because of this, ongoing gene expression in fully grown oocytes and early embryos relies on stored pools of mRNAs that were stockpiled over an extended period of time.

Oocyte meiotic maturation and meiotic spindle formation are driven by the spatially and temporally controlled translation of these stored mRNAs, many of which contain cytoplasmic polyadenylation elements (CPEs). At the heart of oocyte translational regulation is the cytoplasmic polyadenylation element–binding protein (CPEB) family of RNA-binding proteins. CPEB1 orchestrates a sophisticated program of gene expression by silencing CPE-containing mRNAs prior to meiotic maturation and then activating their translation in a signal-dependent manner to trigger the release from prophase I arrest (Richter 2007). Prior to meiotic maturation, CPEB1, together with translational repressors such as poly(A)-specific ribonuclease (PARN), blocks the translation of mRNA targets which include cyclins A and B. A surge of luteinizing hormone released from the pituitary gland initiates a signaling cascade in pre-ovulatory follicles that culminates in CDK1-dependent phosphorylation of CPEB1 (Kunitomi *et al.* 2024). CPEB1 phosphorylation converts it from a repressor to an activator. This occurs by blocking its association with co-repressors such as PARN while stimulating binding to translational co-activators such as cleavage and polyadenylation specificity factor (CPSF). CPEB1 thus recruits co-activators to its targets in a signal-dependent fashion to promote their polyadenylation and translation, triggering meiotic resumption (Richter 2007). Activated CPEB1 becomes enriched at meiotic spindles, along with spindle-associated transcripts (Pascual *et al.* 2021). The enrichment of the translational machinery together with mRNAs encoding spindle components likely ensures sufficient levels of local protein production for the proper formation and function of the meiotic spindle. Tripolar meiotic spindles were observed in CPEB1-deficient mice (Racki & Richter 2006), consistent with a crucial role for CPEB1 in preventing aneuploidy.

Interestingly, human oocyte meiotic spindles are particularly error prone. Live imaging studies of donor human oocytes, following injection of an mRNA encoding GFP-tagged MAP4 to visualize spindle microtubules, found that the majority of human but not mouse meiosis I spindles became transiently unipolar, tripolar, or apolar during meiotic maturation (Holubcova *et al.* 2015), suggesting that the ability of human oocytes to successfully undergo meiotic maturation is perturbed in low-quality oocytes. The transient loss of spindle bipolarity dramatically increases susceptibility to aneuploidy due to chromosomal lagging at anaphase (Silkworth *et al.* 2009). Similar defects in meiotic spindle structure and function were also observed in mouse oocytes from aged animals. Mouse oocytes from aged but not young animals developed meiotic spindles that also became transiently non-bipolar (Nakagawa & FitzHarris 2017).

Why are the meiotic spindles of human and aged mouse oocytes unstable? Elegant reciprocal nuclear transfer experiments established that factors in the oocyte cytoplasm rather than the nucleus were responsible for the development of abnormal meiotic spindles in aged animals, which is consistent with a role for age-associated alterations in translation in driving meiotic spindle instability. Transplantation of nuclei from aged oocytes into the cytoplasm of young oocytes restored their ability to form normal meiotic spindles, whereas young nuclei transplanted into aged oocyte cytoplasm had defective spindles (Nakagawa & FitzHarris 2017). These data show that the failure to maintain spindle bipolarity during meiotic maturation is likely not a consequence of diminished cohesion during prolonged storage of primordial follicles, at least in mouse oocytes. Recent findings from the Conti lab support the hypothesis that reduced translation in late-stage oocytes during ovarian aging is an important contributor to meiotic defects. The Conti lab observed a ~50% decrease in *Cpeb1* mRNA translation in oocytes from aged mice, resulting in lower CPEB1 protein levels (Takahashi *et al.* 2023). This reduction in *Cpeb1* translation was associated with premature de-repression of CPEB1 targets, such as cyclin B1, and an accelerated exit from prophase I arrest in isolated GV stage oocytes. To test the potential effect of diminished CPEB1 production on translation and oocyte function, the Conti lab examined the effect of decreased CPEB1 protein levels in young heterozygous CPEB1^+/–^ mice. They found that these mice had reduced fertility and also produced oocytes with altered translation of CPEB-targets similar to aged wild-type animals (Takahashi *et al.* 2023). Together, these data suggest that diminished translation of even a single factor during aging can cause functional deficits in late-stage oocytes.

Why would oocyte translation be reduced during aging? Very little is known about the stability of ribosomes, tRNAs, and mRNAs undergoing long periods of storage *in vivo*. Data from our group suggests that gene expression using stored mRNAs and ribosomes is much less stable than gene expression utilizing ongoing transcription (Greenblatt *et al.* 2019). We developed methods to delay the ovulation of transcriptionally repressed *Drosophila* mature oocytes arrested in metaphase I for several weeks by controlling the access of females to mates and nutrients. Using this approach, we compared the levels of ongoing translation in mature oocytes stored for various lengths of time with their ability to successfully complete meiosis. We found that the levels of mRNAs in aged *Drosophila* oocytes were generally stable, consistent with findings in aged mouse oocytes (Hamatani *et al.* 2004). In contrast, the global levels of translation went down considerably (>50%) over the course of several weeks of storage. Importantly, the reduction in translation occurring during follicle storage correlated with the appearance of non-bipolar meiotic spindles and the failure of embryos to develop due to meiotic errors. Using ribosome profiling, we measured the levels of translation across all oocyte mRNAs to determine whether the program of translation of meiotic mRNAs was protected from the global decline that occurred during prolonged ovarian storage of oocytes. We observed a substantial loss of translation from mRNAs important for meiotic progression, including mRNAs encoding components of the spindle as well as the spindle assembly checkpoint. Together, these data suggest that oocytes fail to maintain translation of key transcripts during their prolonged storage, and that their reduced levels of translation reflect a pathological decline in ongoing gene expression rather than a shutting-down of translation of non-essential genes. This decline could be caused by a loss of function of any essential translation factor, including ribosomes, tRNAs, mRNAs, etc. A central role for translation decline in limiting oocyte longevity is further supported by our finding that germline-specific loss of the RNA binding protein FMRP, which functions to boost oocyte translation from ~5% mRNAs by ~2-fold, halves the functional lifespan of stored oocytes (Greenblatt & Spradling 2018).

The local ovarian environment of the developing egg changes substantially during aging, as the ovary becomes increasingly fibrotic and stiffer (Amargant *et al.* 2020). The stiffening environment of the ovary could substantially diminish the rate of follicle development, and therefore increase the length of time that oocytes must survive using stored mRNAs, tRNAs, and ribosomes (Fig. 1). The proliferation of aged granulosa cells also appears to diminish over time, slowing down the rate of mouse follicle growth *ex vivo* (Wang *et al.* 2024). There is currently a paucity of data regarding the rates of development of follicles in young vs aged females. In addition, little is known about the functional half-life of mRNAs, tRNAs, and ribosomes *in vivo* in any cell type. Interestingly, oxidative damage to mRNA and rRNA increases dramatically in the motor neurons of amyotrophic lateral sclerosis (ALS) patients (Chang *et al.* 2008), suggesting that damage to the translation machinery may contribute to deficits in diseases of aging such as ALS. Human disease data show that translation dysfunction can lead to premature infertility. Disorders caused by reduced levels of translation factors include Vanishing White Matter (VWM) disease and Diamond-Blackfan anemia (DBA), which are primarily caused by mutations in subunits of the translation initiation factor eIF2B and ribosomal subunits respectively. VWM has been described as an ‘ovarioleukodystrophy’ as female VWM patients frequently present with a loss of motor function together with primary ovarian insufficiency (Parihar *et al.* 2022). Reduced expression of ribosomal subunits in DBA patients leads to a rare blood disorder that disrupts the production of red blood cells, and 75% of female patients with DBA also report menopause by age 40 as a co-morbidity (Tufano *et al.* 2014).

**Figure 1:**
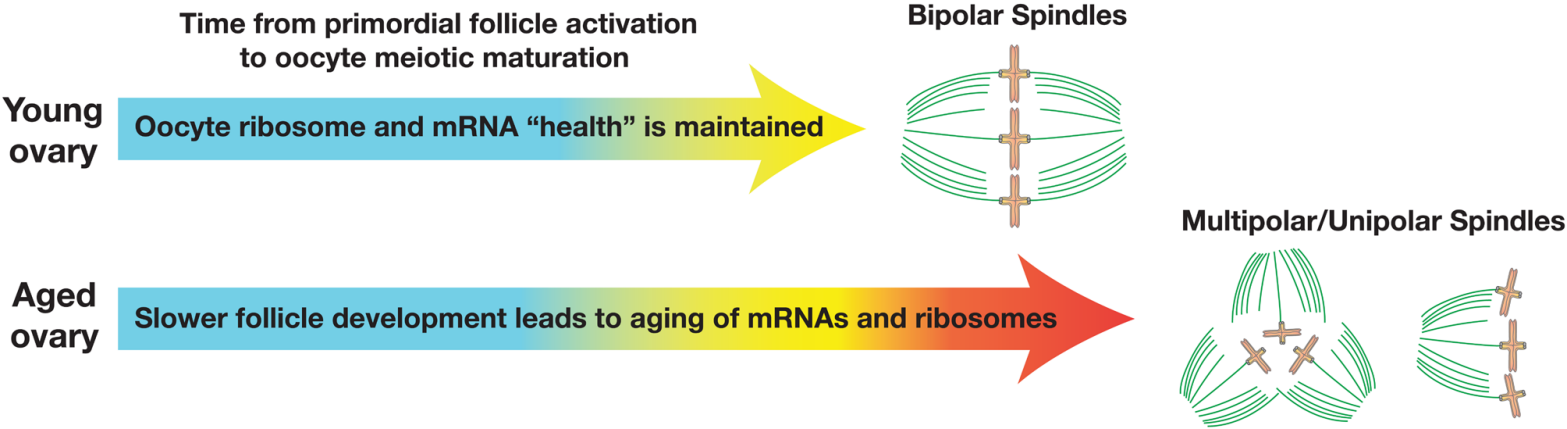
Potential mechanism of oocyte translation decline during ovarian aging. Oocytes in pre-ovulatory follicles utilize mRNAs, tRNAs, and ribosomes that were previously accumulated during follicle development. During aging, follicle development may be slowed by a number of factors, such as the increased stiffness of the ovary, reduced rate of granulosa cell proliferation, limited access to nutrition, etc. This reduced rate of development increases the length of time that the components of the translational apparatus must survive in oocytes, diminishing the functionality of these molecules. The reduced ability of oocytes to support sufficient levels of translation could lead to defects observed during meiotic maturation, including the loss of spindle bipolarity during metaphase I (spindles shown on right).

Could the translation machinery be directly targeted to improve fertility? Recent findings in mice suggest that polyamine supplementation has a protective effect against aging (Liu *et al.* 2017, Zhang *et al.* 2023), and interestingly polyamines have been long known to increase translation rates through multiple mechanisms. The development of markers that identify oocytes with a higher or lower-quality translational machinery may allow for improvements in IVM and IVF protocols and also help identify the most promising oocytes following egg retrieval. We suggest that targeting the translational machinery of the ovary – e.g. through the use of small molecules such as polyamines that directly bind to ribosomes or molecules that improve the quality of the translational apparatus indirectly by improving the rate of follicle development – could represent a new paradigm for the development of therapies for the improvement of fertility.

## Declaration of interest

The authors declare that there is no conflict of interest that could be perceived as prejudicing the impartiality of the study reported.

## Funding

This work was supported by funds from the University of British Columbiahttp://dx.doi.org/10.13039/501100005247 Faculty of Medicine.

## Author contribution statement

KJD, KLJ, and EJG conceptualized the ideas and wrote the manuscript.
